# Supplementary material for: Glycerol-3-phosphate mediates rhizobia-induced systemic signaling in soybean
Source: Nat Commun. 2019 Nov 22;10:5303. doi: 10.1038/s41467-019-13318-8 (PMC6876567; doi:10.1038/s41467-019-13318-8)
Supplement: Supplementary file 1 — Supplementary Information [file 41467_2019_13318_MOESM1_ESM.pdf]

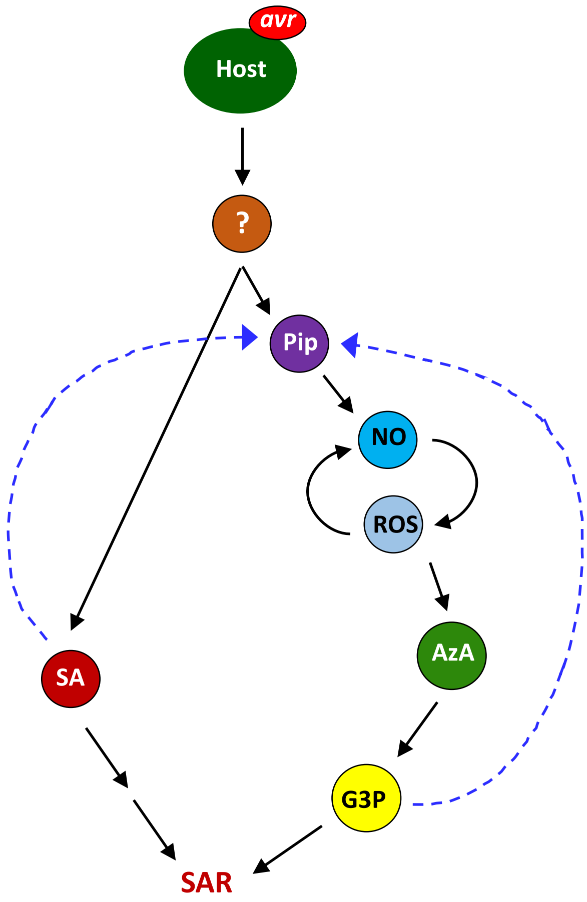

Supplemental Figure 1. Simplified model showing chemical signals involved in systemic acquired resistance (SAR). Infection by avirulent pathogen activates an unknown signal(s) (?) in the local leaf (black arrows), which induces the accumulation of salicylic acid (SA) and pipecolic acid (Pip). Pip induces the accumulation of nitric oxide (NO), which operates in a feedback loop with reactive oxygen species (ROS) to generate azelaic acid (AzA). AzA triggers the biosynthesis of glycerol-3-phosphate (G3P). In the uninfected tissue (dotted blue arrows), G3P and SA are required for Pip biosynthesis, which reinitiates the loop for NO/ROS-AzA-G3P. The SA and G3P-derived pathways operate in parallel to induce SAR.

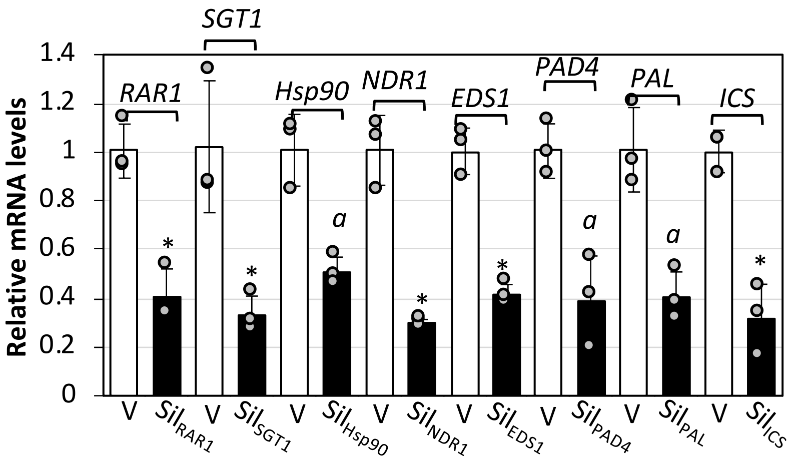

Supplemental Figure 2. Knockdown of conserved *R*-mediated signaling components in *Rj2 rfg1* plants. Relative mRNA levels of indicated genes in V (VIGS control plants) and the respective knockdown lines (Sil_{RAR1}, Sil_{SGT1}, Sil_{Hsp90}, Sil_{NDR1}, Sil_{EDS1}, Sil_{PAD4}, Sil_{ICS1}, Sil_{PAL}) as determined by qRT-PCR. Error bars indicate standard deviation (n=3). Asterisks (P< 0.002) and “a” (P<0.05) denote significant differences (Student’s *t*-test) from respective V.

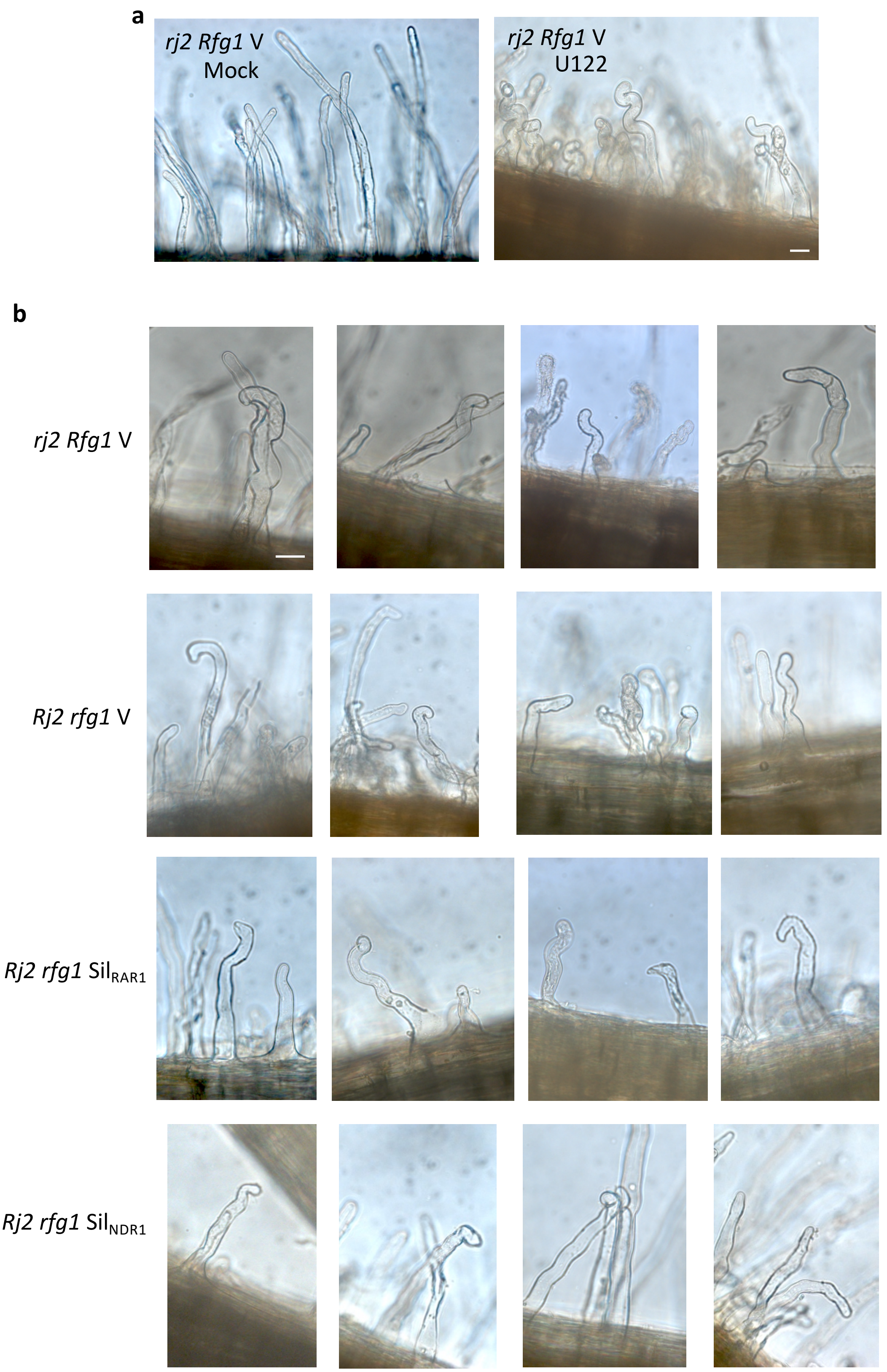

Supplemental Figure 3. Nodulation phenotype of RAR1 and NDR1 knockdown plants. (**a**) Root hair morphology of *rj2 Rfg1* V (VIGS control) inoculated with buffer (Mock) or U122 at 3 dpi. (**b**) Root hair morphology of U122-inoculated *rj2 Rfg1* V, *Rj2 rfg1* V, *Rj2 rfg1* Sil$_{RAR1}$ (RAR1-knockdown) and *Rj2 rfg1* Sil$_{NDR1}$ (NDR1-knockdown) soybean plants. Transmission micrographs were taken at 3 days post inoculation with U122. Scale bars represent 270 microns. Results are representative of two independent experiments.

a

Gene silenced	*Rj2 rfg1* U122 (I)	*Rj2 rfg1* U257 (C)	*rj2 Rfg1* U257 (I)	*rj2 Rfg1* U122 (C)	*rj2 rfg1* U122 (C)
Mock	0	41 (+/-9)	0	20 (+/-10)	36 (+/-12)
V	0	30 (+/-15)	0	24 (+/-17)	38 (+/-6)
RAR1	26 (+/-8)	28 (+/-7)	23 (+/-5)	19 (+/-9)	32 (+/-5)
SGT1	0	33 (+/-11)	0	26 (+/-11)	41 (+/-13)
Hsp90	0	30 (+/-10)	0	28 (+/-8)	30 (+/-4)
NDR1	30 (+/-10)	29 (+/-9)	21 (+/-10)	17 (+/-12)	48 (+/-17)
EDS1	0	32 (+/-10)	0	26 (+/-12)	39 (+/-5)
PAD4	0	25 (+/-11)	0	24 (+/-9)	44 (+/-11)

b

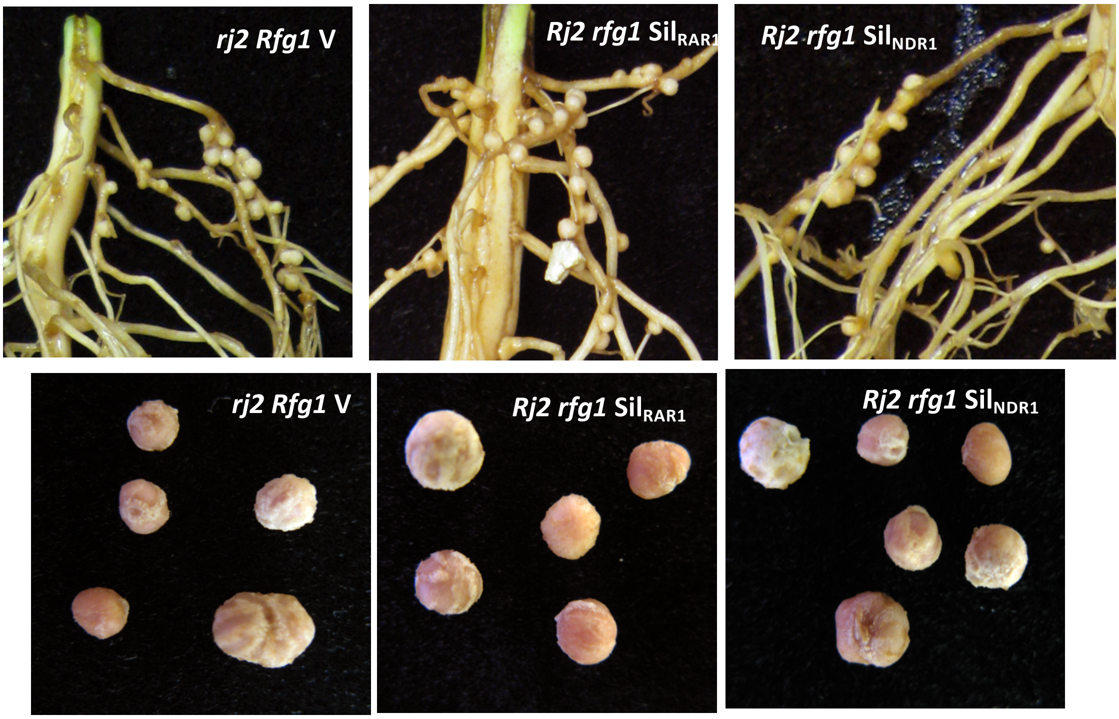

Supplemental Figure 4. Knockdown of conserved *R*-mediated signaling components in *Rj2 rfg1* plants. (**a**) Table showing average number of nodules/plant produced by USDA122 (U122) or USDA257 (U257) in V (VIGS control plants) and the respective knockdown lines (Sil$_{RAR1}$, Sil$_{SGT1}$, Sil$_{Hsp90}$, Sil$_{NDR1}$, Sil$_{EDS1}$, Sil$_{PAD4}$, Sil$_{ICS1}$, Sil$_{PAL}$) in the indicated background. +/- indicates standard deviation (n=10-15 plants). "I" and "C" indicate incompatibility or compatibility between plant genotype and rhizobium strain, respectively. (**b**) Density of nodules on roots (upper panels) of *rj2 Rfg1* V, *Rj2 rfg1* Sil$_{RAR1}$ and *Rj2 rfg1* Sil$_{NDR1}$ plants at 2 weeks post inoculation with U122. and morphology of nodules (lower panels) produced on *rj2 Rfg1* V (VIGS control) , *Rj2 rfg1* Sil$_{RAR1}$ (RAR1-knockdown) and *Rj2 rfg1* Sil$_{NDR1}$ (NDR1-knockdown) plants at 3 weeks post inoculation with U122. Results are representative of three-four independent experiments.

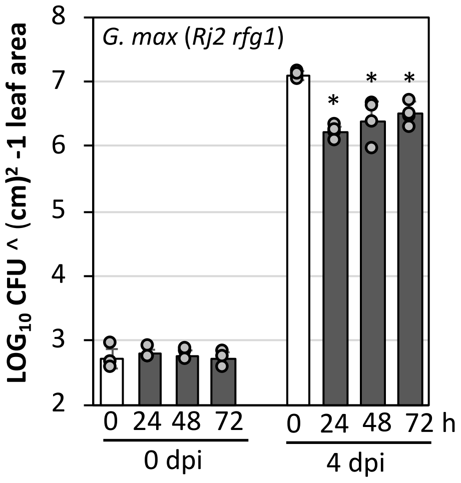

Supplemental Figure 5. Effectiveness of rhizobia-induced systemic immunity. Bacterial counts of *Psg Vir* in *Rj2 rfg1* plants pretreated (root inoculation) with U122. *Psg Vir* was leaf-infiltrated at 0, 24, 48 or 72 h post U122 application. LOG_{10} values of colony forming units (CFU) per cm^2 leaf area from infected leaves at 0 and 4 days post-inoculation (dpi) are presented. Error bars indicate standard deviation (n=5). Asterisks denote data significantly different from 0 h samples, Student's *t*-test, P<0.001. Results are representative of three independent experiments.

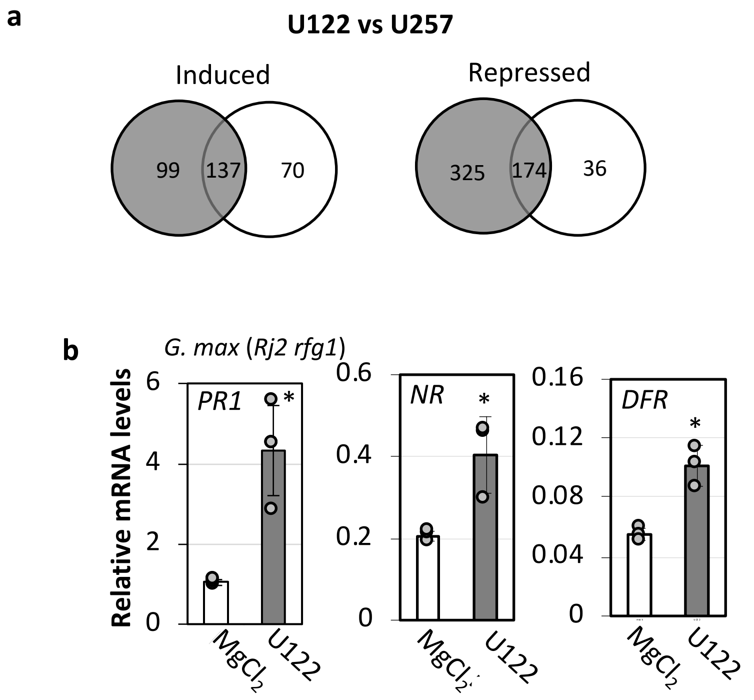

Supplemental Figure 6. Root inoculation with incompatible rhizobia induces distinct transcriptional changes in the leaf tissue of soybean plants. (**a**) Venn diagrams showing the number of genes induced or repressed in the foliar tissue of soybean plants inoculated with U122 versus U257. (**b**) Relative mRNA levels of *PR1, NR,* and *DFR* in the leaves of *Rj2 rfg1* plants treated with buffer ($MgCl_2$) or incompatible rhizobia (U122), as determined by qRT-PCR. Error bars indicate standard deviation (n=3). Asterisks denote significant differences from Mock, Student's *t*-test, P< 0.005.

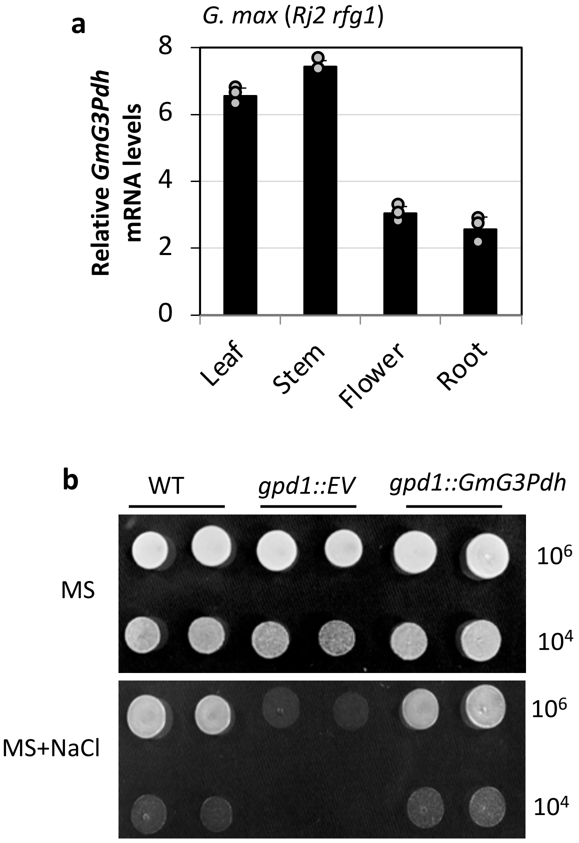

Supplemental Figure 7. Soybean G3Pdh encodes a functional G3Pdh enzyme. (**a**) Quantitative RT-PCR analysis showing relative levels of *GmG3Pdh* in indicated soybean tissues. (**b**) Yeast complementation assay showing complementation of the yeast *gpd1* mutation by *GmG3Pdh* gene. Unlike wildtype yeast cells (WT), the *gpd1* mutant (defective in yeast G3Pdh) expressing empty vector (*gpd1*::*EV*) is unable to grow on synthetic media containing NaCl. Wild-type-like growth is restored in *gpd1* expressing GmG3Pdh (*gpd1*::*GmG3Pdh*). Results are representative of two independent experiments.

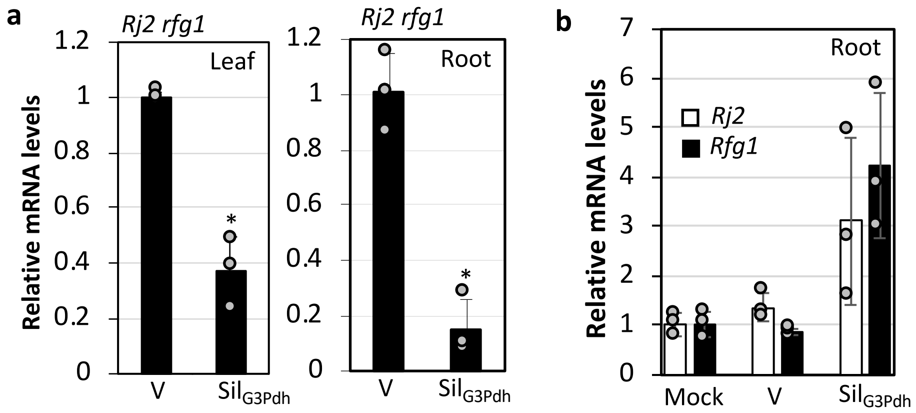

Supplemental Figure 8. Silencing G3Pdh in soybean. (**a**) Quantitative RT-PCR analysis showing relative mRNA levels of *GmG3Pdh* in leaf and root tissue of *Rj2 rfg1* plants infected with the control VIGS vector (V) or *GmG3Pdh* knock down plants (Sil_{G3Pdh}) plants. (**b**) Quantitative RT-PCR analysis showing relative mRNA levels of *Rj2* and *Rfg1* in root tissue of mock-infected (Mock), V, and Sil_{G3Pdh} plants. Error bars indicate standard deviation (n=3). Asterisks denote significant differences from Mock, Student's *t*-test, P< 0.0001. Results are representative of two independent experiments.

a

Genotype	Root hair curling/ cm root	Total no root hair/cm root	Nodule primordia/ cm root
rj2 Rfg1	1.55 (+/-0.83)	147 (+/-18)	0.6 (+/-0.09)
Rj2 rfg1 V	1 (+/-0.63)	181 (+/-16)	0.12 (+/-0.09)
Rj2 rfg1 Sil_{G3Pdh}	1.33 (+/-0.82)	166 (+/-22)	0.53 (+/-0.11)

b

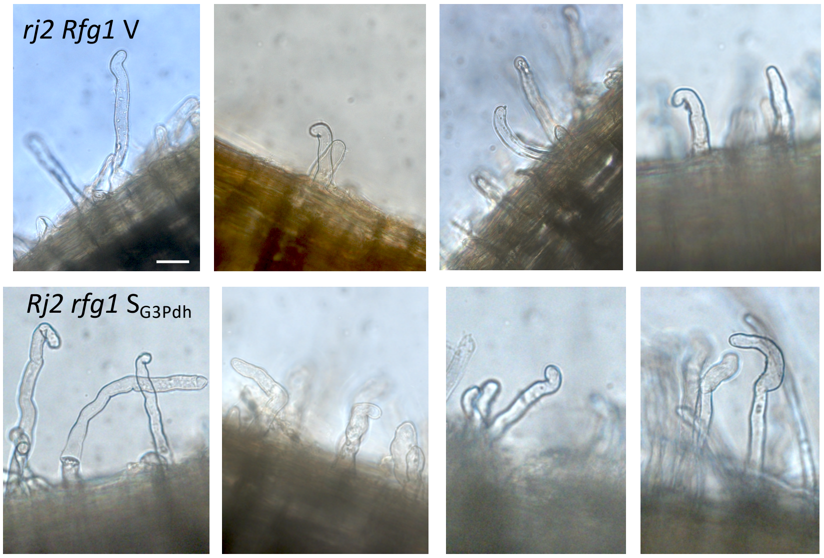

c

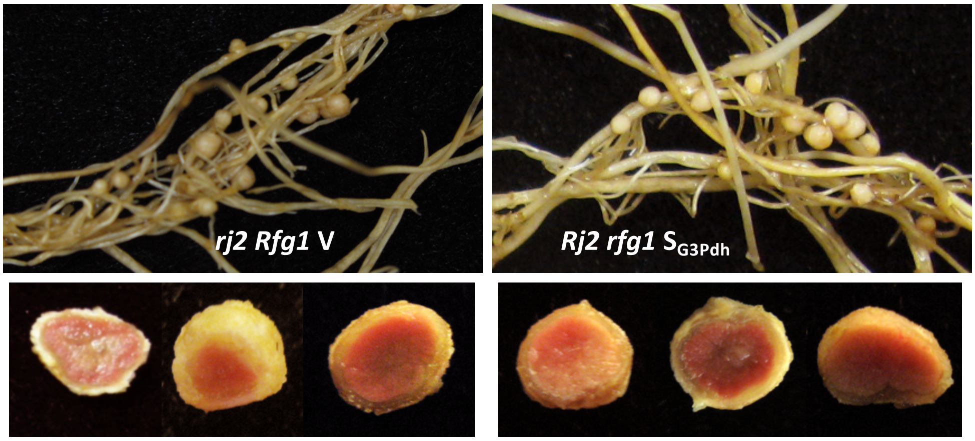

Supplemental Figure 9. Effect of silencing G3Pdh in soybean. (**a**) Table showing number of infection pockets (3 dpi) and nodule primordia (6 dpi) in U122 inoculated *rj2 Rfg1* V (VIGS control), *Rj2 rfg1* V and *Rj2 rfg1* Sil_{G3Pdh} (G3Pdh-knockdown) plants. (**b**) Root hair morphology of U122-inoculated *rj2 Rfg1* V and *Rj2 rfg1* Sil_{G3Pdh} soybean plants. Transmission micrographs were taken at 3 days post inoculation with U122. Scale bars represent 270 microns. (**c**) Density (upper panels) and morphology (lower panels, cut nodules are shown) of nodules on roots of *rj2 Rfg1* V and *Rj2 rfg1* Sil_{G3Pdh} plants at 2-3 weeks post inoculation with U122. Results are representative of two-three independent experiments.

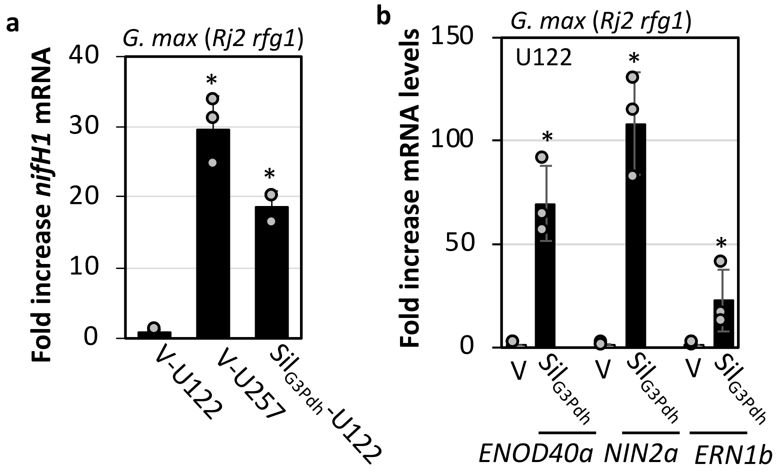

Supplemental Figure 10. Soybean G3Pdh contributes to *Rj2*-mediated exclusion of incompatible rhizobia. (**a**) Quantitative RT-PCR analysis showing fold increase in the levels of bacterial *nifH1* mRNA in root tissue of V (VIGS control) or $\mathrm{Sil}_{\mathrm{G3Pdh}}$ (*GmG3Pdh* knockdown) *Rj2 rfg1* plants inoculated with indicated strain. (**b**) Quantitative RT-PCR analysis showing fold increase in plant *ENOD40a, NIN2a, ERN1b* mRNA in root tissue of U122 inoculated $\mathrm{Sil}_{\mathrm{G3Pdh}}$ *Rj2 rfg1* plants. Error bars indicate standard deviation (n=3). Asterisks denote significant differences from V-U122 in **a** and **b**, Student's *t*-test, P< 0.0005. Results are representative of two independent experiments.

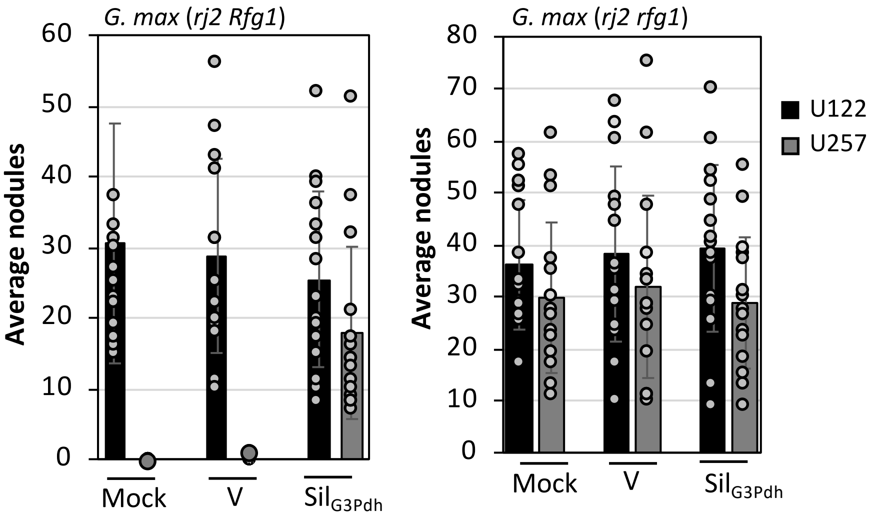

Supplemental Figure 11. G3P does not contribute to compatible soybean-rhizobium interactions. (Left panel) Average number of nodules produced per plant by compatible and incompatible rhizobia in *Rfg1 rj2* plants that were mock inoculated (Mock), infected with the control VIGS vector (V), or knocked down for *GmG3Pdh* (Sil_{G3Pdh}). USDA 122 (U122, black bars) is compatible with *Rfg1 rj2* plants, while USDA257 (U257, produced by U122 or U257 on Mock, V or Sil_{G3Pdh} plants in grey bars) is incompatible. (Right panel) Average number of nodules *rj2 rfg1* background. Error bars indicate SD (n=12-15). Grey circles on *x* axis indicates absence of nodules. Results are representative of three-four independent experiments.

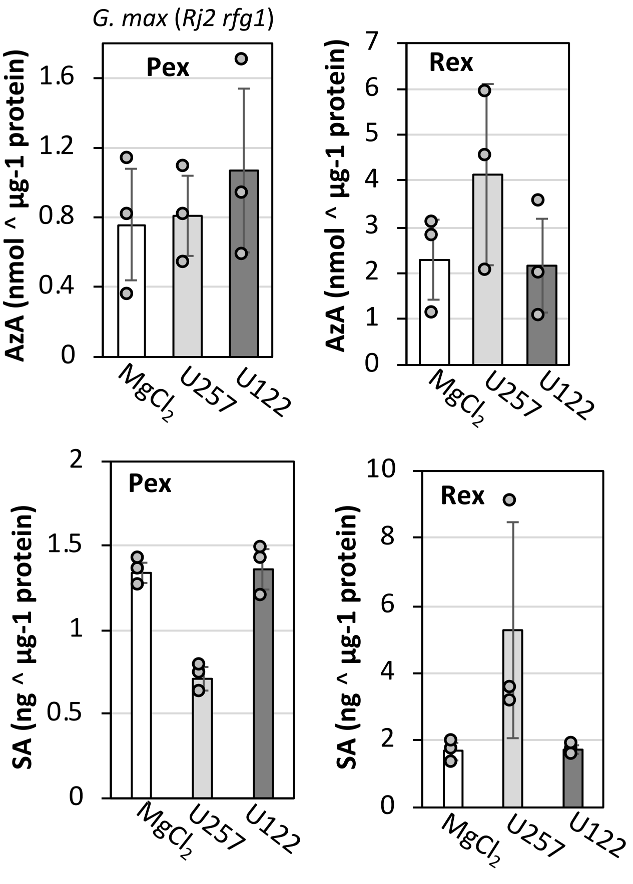

Supplemental Figure 12. AzA and SA do not accumulate in response to incompatible rhizobia. AzA and SA levels in leaf (Pex) or root (Rex) exudates of *Rj2 rfg1* plants inoculated with buffer ($MgCl_2$), compatible (U257) or incompatible (U122) rhizobia. Error bars indicate standard deviation (n=3). Results are representative of two-three independent experiments.

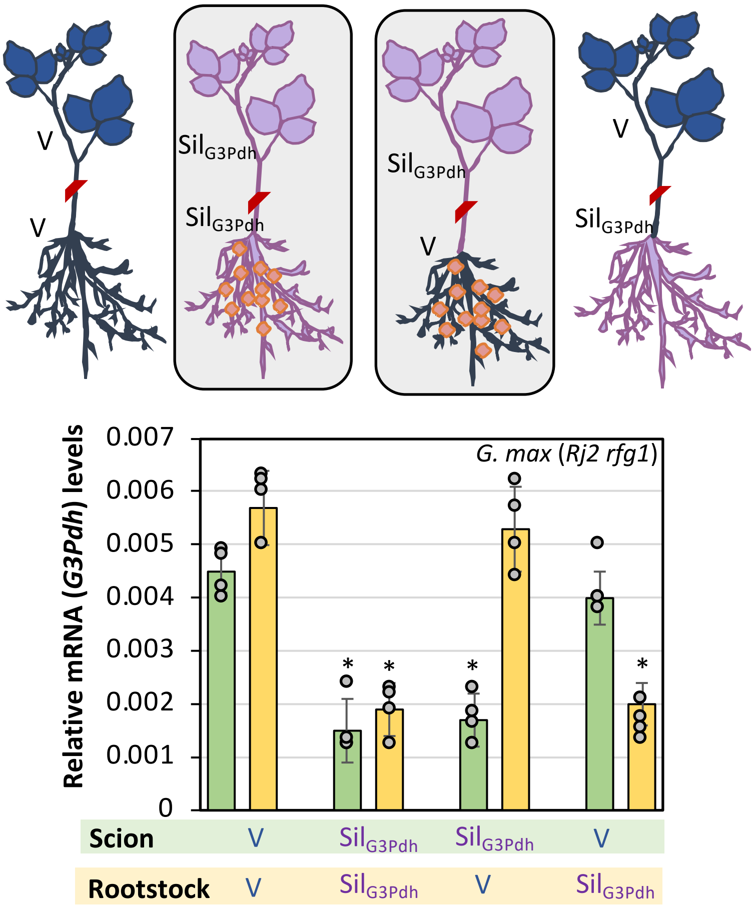

Supplemental Figure 13. Quantitative RT-PCR analysis showing relative mRNA of *GmG3Pdh* in scion and rootstock of *Rj2 rfg1* V and *Rj2 rfg1* Sil$_{G3Pdh}$ grafts. V=plants infected with the control VIGS vector, Sil$_{G3Pdh}$=plants knocked down for *GmG3Pdh.* Error bars indicate standard deviation (n=4). Asterisks denote significant differences from VxV grafts, Student's *t*-test, P< 0.001. Boxes indicate grafts that produced nodules in response to the incompatible U122. Results are representative of three independent experiments.

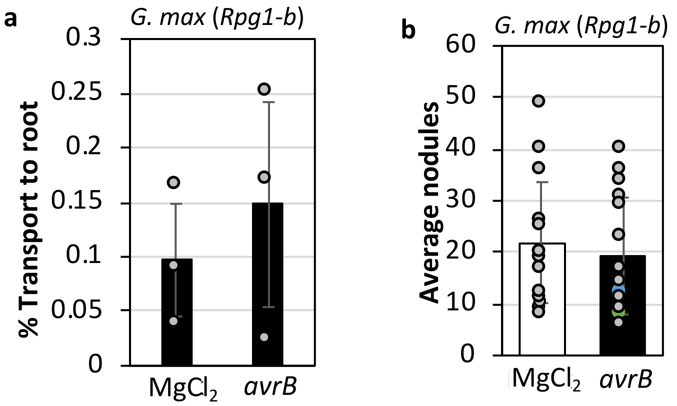

Supplemental Figure 14. Pathogen infection does not promote G3P transport to the root. (**a**) Percentage of ^{14}C transported to root in plants (*Rpg1-b*) that were leaf-infiltrated with ^{14}C-G3P along with buffer ($MgCl_2$) or *Psg avrB* (*avrB*). ^{14}C levels were measured in root extract 48 h after *avrB* inoculation. (**b**) Average nodules per plant produced by U122 on $MgCl_2$-infiltrated or *avrB*-infected *Rpg1-b* plants. Error bars indicate standard deviation (n=15). Results are representative of two independent experiments.

Supplementary Table 1: Primer List

**Primers for qPCR**	
Actin Fwd	GAGCTATGAATTGCCTGATGG
Actin Rev	CGTITCATGAATTCCAGTAGC
DFR Fwd	ATTGTCTCTGTGGAAGGCTG
DFR Rev	TTTTCCCCTCAGTGTCCTTG
EDS1 Fwd	ATGGCTGGAGGGTTGCTTG
EDS1 Rev	CATTCAGCGGGATCCCAAGAT
ENOD40a Fwd	TGGAGTCCATTGCCTTTTCG
ENOD40a Rev	TCTCTCTTGAGTGGCAGAAGCA
ERN1b Fwd	TGTTGGAGTGAGACAAAGGC
ERN1b Rev	GAAGTTGGTGCGAGTGTTTG
G3PDH Fwd	GCTTCTCCTTCTCTTCCTCAAG
G3PDH Rev	AGGCAGTAATCAGCACCAAG
HSP90 Fwd	AGGCCGAGATCAACCAGCTCCTC
HSP90 Rev	GTCATGCCAATACCACTGTCAA
ICS Fwd	TCACTCATTTCACCGACACC
ICS Rev	TGAATATTGGTGCTGTCCCTC
NDR1 Fwd	CTCGGCAACGCCACCGTGGAA
NDR1 Rev	GGACTCGATCACCACGGGGTTC
NIN2a Fwd	CACAACCTCTTCCTCAACTCC
NIN2a Rev	CTTTCCCATTGTCCTTGTGTTG
NR Fwd	AGTACGGAAAAGATGGGTGTG
NR Rev	CTAGTCAATCCAACCTCATCGAG
PAD4 Fwd	CGCGGATCCTGGCACTTGTCCATGACT
PAD4 Rev	GGAGCAGTGTGTGTGGATAGTCC
PAL Fwd	AGAATGCAGATCTTACCCACTG
PAL Rev	TTGTCAAACTCTTCACCCGG
PR1 Fwd	ATGGGGTACATGTGCATTAAG
PR1 Rev	CTACAGTTTGTAGGGTCTTTC
RAR1 Fwd	GGATTGGGTGCGACGCCATG
RAR1 Rev	GGTGTTCTTCTTCACTGGTGT
Rj2 Fwd	CTTCAAGCATTAGAACTGCGC
Rj2 Rev	TCACCCTCTTCCTGTTTTAGC
Rfg1 Fwd	CTTCAAGCATTAGACCTGAGC
Rfg1 Rev	GGCACCTCATACGGACTGTT
SGT Fwd	CAGAAGCCAGAAGAAGTGGT
SGT Rev	GATGGGTATGAAGCCCTTTCAG
**Primers for generating GmG3Pdh knockdown vector**	
G3Pdhsil BamHI Fwd	GCATGGATCCGGGATAGTAGAAGGTATG
G3Pdhsil MscI Rev	CAGTTGGCCATGTAAGCATTATGTCTCC
