# Supplementary material for: Glycerol-3-phosphate mediates rhizobia-induced systemic signaling in soybean
Source: Nat Commun. 2019 Nov 22;10:5303. doi: 10.1038/s41467-019-13318-8 (PMC6876567; doi:10.1038/s41467-019-13318-8)
Supplement: Supplementary file 3 — Description of Additional Supplementary Files [file 41467_2019_13318_MOESM3_ESM.pdf]

Description of Additional Supplementary Files

File Name: Supplementary Data 1
Description: Transcript levels of genes induced (two fold or higher, p value ≤ 0.05) in foliar tissues of soybean plants inoculated with incompatible strain (U122) of rhizobia compared to compatible strain (U257).

File Name: Supplementary Data 2
Description: Transcript levels of genes repressed (two fold or less, p value ≤ 0.05) in foliar tissues of soybean plants inoculated with incompatible strain (U122) of rhizobia compared to compatible strain (U257).

File Name: Supplementary Data 3
Description: Transcript levels of genes induced (two fold or higher, p value ≤ 0.05) in foliar tissues of soybean plants inoculated with incompatible strain (U122) of rhizobia compared to mock-inocualted plants.

File Name: Supplementary Data 4
Description: Transcript levels of genes repressed (two fold or lower, p value ≤ 0.05) in foliar tissues of soybean plants inoculated with incompatible strain (U122) of rhizobia.

File Name: Supplementary Data 5
Description: Transcript levels of genes induced (two fold or higher, p value ≤ 0.05) in foliar tissues of soybean plants inoculated with compatible strain (U257) of rhizobia.

File Name: Supplementary Data 6
Description: Transcript levels of genes repressed (two fold or higher, p value ≤ 0.05) in foliar tissues of soybean plants inoculated with compatible strain (U257) of rhizobia.
